# Changes in DNA methylation in naïve T helper cells regulate the pathophysiological state in minimal-change nephrotic syndrome

**DOI:** 10.1186/s13104-017-2719-1

**Published:** 2017-09-15

**Authors:** Yasuko Kobayashi, Akira Aizawa, Takumi Takizawa, Katsuhide Igarashi, Izuho Hatada, Hirokazu Arakawa

**Affiliations:** 1 Department of Pediatrics, Gunma University Graduate School of Medicine, 3-39-22 Showa-machi, Maebashi, Gunma, 371-8511 Japan; 2 Life Science Tokyo Advanced Research Center (L-StaR), Hoshi University School of Pharmacy and Pharmaceutical Sciences, Tokyo, Japan; 3 Laboratory of Genome Science, Biosignal Genome Resource Center, Gunma University Institute for Molecular and Cellular Regulation, Maebashi, Japan; 4 Center for Fiber and Textile Science, Kyoto Institute of Technology, Matsugasaki, Kyoto, Japan

**Keywords:** Nephrotic syndrome, Minimal-change, Children, DNA methylation, Microarray-based integrated analysis of methylation by isoschizomers method, Helper T cell, Monocyte

## Abstract

**Background:**

DNA methylation plays a crucial role in regulating transcription, and changes in DNA methylation affect gene expression and disease development. Minimal change nephrotic syndrome (MCNS) has been reported to involve immunological disturbances. Since the characteristic features of the disease include recurrent relapse and sex and age preference, the disease pathogenesis may be partly related to epigenetic changes. However, little is known about these changes.

**Methods:**

We analyzed genome-wide DNA methylation using the microarray-based integrated analysis of methylation by isoschizomers method. This method was used to evaluate methylation in monocytes (patient number; n = 6) and naïve T helper cells (n = 4) from the peripheral blood of MCNS patients both in relapse and following remission and that of healthy controls (n = 5).

**Results:**

In total, 85 co-occurring genes were identified in naïve T helper cells, while 4 such genes were identified in monocytes, which were common among the 3 following comparisons for changes in DNA methylation using sample pairs: (1) relapse versus remission, (2) relapse versus controls, and (3) remission versus controls. In 82 of 85 co-occurring genes (96.5%) in naïve T helper cells, the level of DNA methylation was altered according to disease activity, but was not related to disease activity in the 4 genes detected in monocytes.

**Conclusions:**

Therefore, in 82 co-occurring genes in naïve T helper cells, the regulation of DNA methylation was well correlated with the clinical and pathophysiological state. Our genome-wide approach to analyze DNA methylation provides further insight into the pathogenesis of MCNS and indicates potential prediction and diagnostic tool for the disease.

**Electronic supplementary material:**

The online version of this article (doi:10.1186/s13104-017-2719-1) contains supplementary material, which is available to authorized users.

## Background

Minimal-change nephrotic syndrome (MCNS) is the most common cause of nephrotic syndrome in children. It is characterized by massive proteinuria and hypoalbuminemia in a relapse/remission course without histological evidence of immune-mediated inflammatory damage. These manifestations are typically reversible with corticosteroid therapy. Although the pathogenesis of MCNS remains unknown, immunological disruption has been implicated in this disease [1], and T cell-derived vascular permeability factors have been shown to be responsible for alterations in glomerular permeability [2–4]. During childhood, the incidence of MCNS has been reported to be twofold higher in male children, with a prevalence that is inversely proportional to age. Recurrent relapse tends to decrease after adolescence [5, 6]. The characteristic features of MCNS include a recurrent relapse/remission course, gender preference, age preference of onset and relapse, and steroid response in most patients, therefore, a single genetic defect is likely not responsible for the disease. However, epigenetic alterations may occur without a direct change in the genetic sequence and regulate the phenotype in relapsed MCNS patients.

Epigenetics is the study of mitotically heritable changes, which alter gene expression without direct DNA sequence alterations. DNA methylation, one of the principal epigenetic mechanisms in mammals, involves the covalent addition of a methyl group to a cytosine residue followed by a guanine residue [7]. DNA methylation regulates gene expression and is essential for differentiation, embryonic development [8], genomic imprinting [9], and X-chromosome inactivation [10]. DNA methylation within the promoter region of a gene is commonly associated with transcriptional inactivation, whereas demethylation contributes to transcriptional activation. Changes in the DNA methylation profile can also lead to differences in gene expression programs and thus influence disease development [11].

Audard et al. reported that nuclear factor related to kappa B binding protein (NFRKB) is highly expressed in the nuclear compartment during relapse and that NFRKB promotes the hypomethylation of genomic DNA, suggesting epigenetic involvement in the pathogenesis of MCNS [12]. Elie et al. suggested that alterations in epigenetic modifications induced by external cues such as viral infection affect MCNS progression; this is supported by the fact that MCNS relapse is frequently triggered by external or internal environmental factors. Such environmental changes affect the epigenotype and alter gene expression [13]. We previously reported that the DNA methylation states of 3 genes, GATA binding protein 2 (*GATA2*), pre-B cell leukemia homeobox 4 (*PBX4*), and nyctalopin (*NYX*) in naïve T helper cells (Th0s), but not in monocytes, significantly differed between relapse and remission in affected patients [14]. Additionally, epigenetic regulation in Th0s underlies the pathogenesis of MCNS, the disturbance of which has been implicated in MCNS development [14].

In this study, we examined whether DNA methylation states change according to the clinical states of MCNS. We identified candidate genes whose DNA methylation states change during relapse and remission and compared them to those in healthy controls to gain insight into MCNS pathogenesis.

## Results

### Study population characteristics

The mean total follow-up period for patients until December 2012 was 122.2 months (range 53–204 months). All patients were steroid-responsive and experienced frequent relapses. The mean total relapse time was 8.3 (range 4–15 times) during the follow-up period. Although it has been already known that the likelihood that a child with primary nephrotic syndrome who responds during eight weeks of intensive initial steroid treatment has MCNS is quite high and that renal biopsy need not be performed as prognosis in these patients is considered to be very favorable [15], renal biopsy was performed for histological evaluation of the side effects of cyclosporine in 4 patients who were administered with cyclosporine for frequent relapse during the follow-up periods. The histological findings were consistent with those of MCNS. The mean age at sampling in relapse was 13 years 5 months (range 6–19 years and 10 months) and the mean number of relapses until sampling was 6 (range 3–13 relapses) (Table 1). The mean sampling interval from relapse to subsequent remission was 25 weeks (range 5–71 weeks). The therapeutic conditions were similar at sampling for relapse and remission in all subjects except for patients 2 and 6, who received a corticosteroid (patients 2 and 6) and an immunosuppressant (patient 2) at remission, but not at relapse sampling.

**Table 1:** MCNS patient characteristics at sampling

Pt. no.	Sex	Age at onset	Total follow-up period^a^, months	Total relapse time^a^	FR	Biopsy	Sampling age at rel	Relapse times at sampling	Intervals^b^, weeks	PSL at rel^c^ mg/day	PLS at rem^c^ mg/d	IS at rel^c^ mg/day	IS at rem^c^ mg/day	CD14+, n = 6	RO−, n = 4
1	M	10 years, 4 months	96	6	Yes	MC	13 years, 0 months	3rd	5	40	10	0	0	Taken	Taken
2	M	5 years, 4 months	53	6	Yes	MC	6 years, 0 months	3rd	16	0	35	0	CyA90	Taken	Taken
3	M	5 years, 11 months	204	11	Yes	MC	18 years, 6 months	9th	23	10	15	0	0	Taken	Taken
4	M	7 years, 3 months	192	15	Yes	MC	19 years, 10 months	13th	23	60	5	0	0	Taken	Taken
5	M	9 years, 9 months	109	4	Yes	ND	11 years, 7 months	4th	12	0	0	CyA180	CyA160	Taken	NT
6	M	11 years, 11 months	79	8	Yes	ND	12 years, 5 months	4th	71	0	20	MZV125	MZV 75	Taken	NT
Mean		8 years, 4 months	122.2	8.3			13 years, 5 months	6th	25						

Patients for DNA methylation analysis

*FR* frequent relapser, *MC* minimal change, *ND* not done, *Rel* relapse, *Rem* remission, *PSL* prednisolone, *IS* immunosuppressant, *CyA* cyclosporin A, *MZB* mizoribine, *NT* not taken

^a^Follow-up period until December 2012

^b^Intervals of sampling between relapse and remission

^c^Treatment at the time of sampling

### Microarray-based integrated analysis of methylation by isoschizomers (MIAMI)

**Fig. 1:**
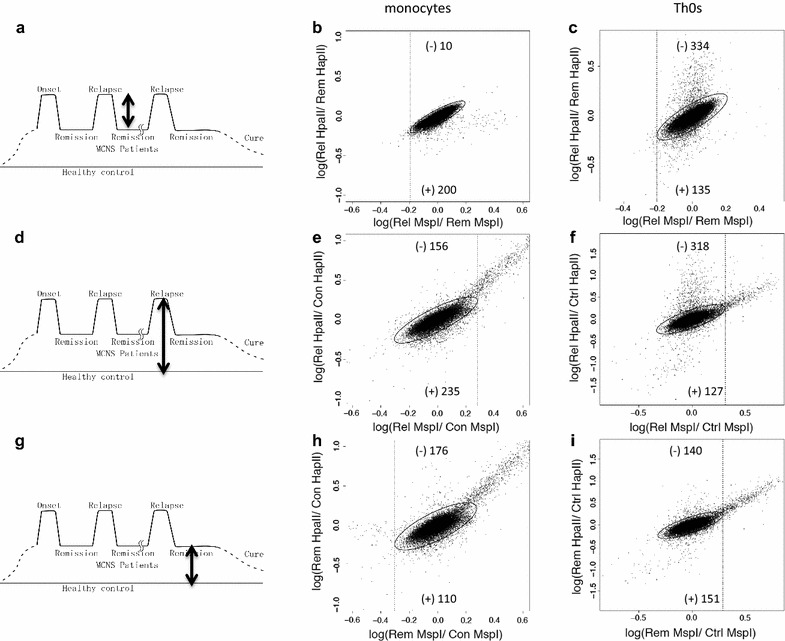
Three sets of comparisons with microarray-based integrated analysis of methylation by isoschizomers (MIAMI). The 3 sets of comparisons are indicated schematically: comparison between **a** relapse and remission, **d** relapse and controls, and **g** remission and controls. Scatter plots of the signals obtained with each probe in MIAMI analysis are represented; comparisons between relapse and remission in monocytes (**b**) and in naïve T helper cells (Th0s) (**c**), between relapse and controls in monocytes (**e**) and in Th0s (**f**), and between remission and controls in monocytes (**h**) and Th0s (**i**) are shown. The y-axis represents the signal intensity ratios of each probe between 2 samples digested with *Hpa*II, a methyl-sensitive restriction enzyme that indicates the differences in DNA methylation between the 2 samples. The x-axis represents the ratios between the same set of samples digested with *Msp*I, which is a methyl-insensitive isoschizomer of *Hpa*II. Thus, the x-axis indicates the digestion efficiency or accessibility of restriction enzymes. For statistical analysis, we used the Mahalanobis distance, which is a simplistic approach for estimating the standard deviation of distance between each sample point and the center of mass. The threshold values were determined at a 99% CI of the Mahalanobis distance from the center of the mass (*solid line*) and 99% CI of the *Msp*I treatment signal plotted on the x-axis (*dash line*). The probes in the upper area of the 99% CI of Mahalanobis distance and concomitantly within 99% CI of *Msp*I digestion corresponded to significantly less methylated in relapse (**b**, **c**, **e**, and **f**) and in remission (**h** and **i**) than each comparative. In contrast, the probes in the lower area indicated significantly more methylated in the relapse (**b**, **c**, **e**, and **f**) or in remission (**h** and **i**). The *numbers in each figure* indicate detected probe numbers in each area; (*minus*) indicates less methylated probes, while (*plus*) indicates more methylated probes. The number of probes detected is shown in Table 2

We first investigated differences in genome-wide DNA methylation between relapse and remission of MCNS patients (Fig. 1a–c), between relapse and controls (Fig. 1d–f), and between remission and controls (Fig. 1g–i), both in monocytes (Fig. 1b, e, h) and in Th0s (Fig. 1c, f, i). For each comparison, the numbers of probes with significant changes in DNA methylation have been shown in Table 2. The MIAMI results were confirmed using bisulfite-pyrosequencing analysis as previously reported [14].

**Table 2:** Number of probes in which DNA methylation changed significantly

Comparisons	Monocytes		Th0s	
	Less methylated	More methylated	Less methylated	More methylated
Relapse versus remission	10	200	334	135
Relapse versus control	156	235	318	127
Remission versus control	176	110	140	151

**Fig. 2:**
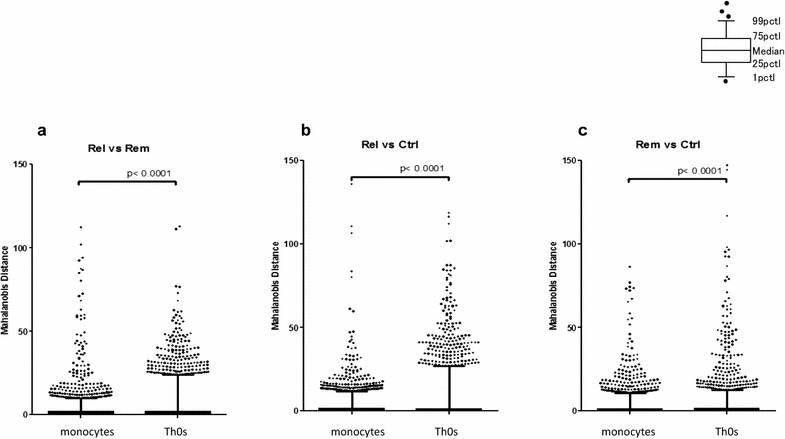
Distance distributions between monocytes and naïve T helper cells (Th0s) in the 3 comparisons. The distance distributions of all probes from the center of the mass were significantly different (*P* < 0.0001) between monocytes and Th0s in each of the 3 sets of comparisons: comparison between **a** relapse and remission, **b** relapse and controls, and **c** remission and controls

For all the probes, the Mahalanobis distance distributions were significantly different (*P* < 0.0001) between monocytes and Th0s in all comparisons (Fig. 2a–c). These results indicate that the regulation of DNA methylation differs between monocytes and Th0s in all comparisons. Specifically, regulation differed between relapse and remission, even in the same individuals (Fig. 2a), as we previously reported [14], as well as between patients both in relapse and in remission and healthy controls (Fig. 2b, c).

### Genes co-occurring among three comparisons in monocytes and Th0s derived from patients in relapse and remission, and healthy controls

**Fig. 3:**
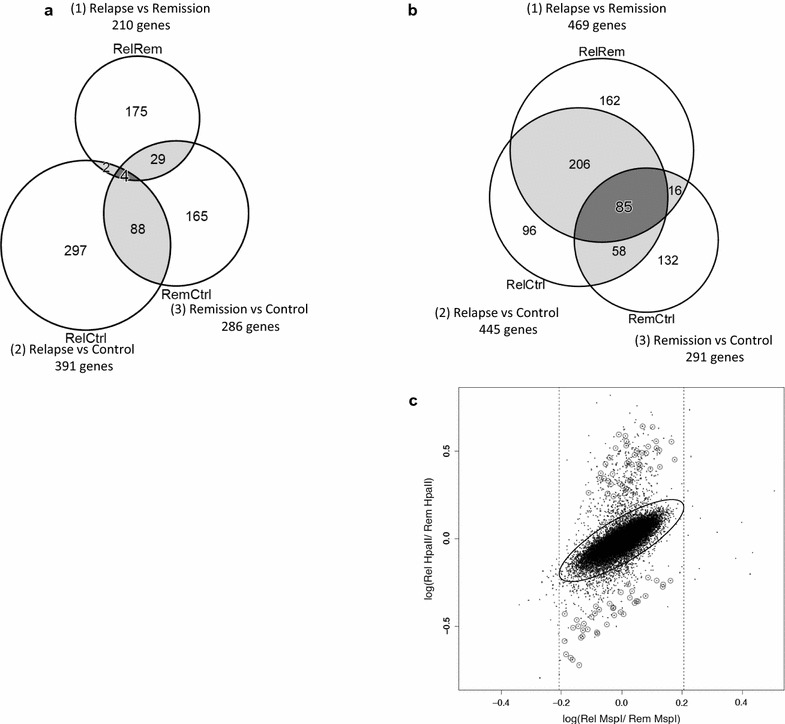
Co-occurring genes* among the 3 comparisons in monocytes and Th0s. The number of probes plotted outside the 99% CI of the Mahalanobis distance within *Msp*I 99% CI among the 3 comparisons are shown in a Venn diagram for monocytes (**a**) and Th0s (**b**). There were 85 co-occurring genes in Th0s in the 3 comparisons and 4 in monocytes. **c** The probes of the 85 co-occurring genes are indicated in the *scatter plots* comparing relapse and remission as a representative of 3 comparisons. The distributions of the 85 probes were equitable and appropriate. *“Co-occuring” genes are those identified to be common among all 3 comparisons

We investigated whether there are probes in monocytes (Fig. 3a) and Th0s (Fig. 3b) that could be detected to be common among the 3 comparisons. The number of genes in Th0s that were identified in all 3 comparisons was 85 (Fig. 3b); however, only 4 genes in monocytes were identified to be common among all 3 comparisons (Fig. 3a). In this paper, we refer to these genes as ‘co-occurring’ genes. The distribution of the 85 probes was not skewed, indicating that the results were not affected by technical bias (Fig. 3c). Further analysis of the DNA methylation status in these genes revealed that 82 of 85 co-occurring genes in Th0s (Table 3) showed changes in methylation according to disease activity.

**Table 3:** List of the 82 genes co-occurring among the three sets of comparisons in Th0 cells

Less methylated in relapse		More methylated in relapse	
Common gene name	Chromosome	Common gene name	Chromosome
C1orf63	1p36.13–p35.1	B3GALT6	1p36.33
ELOVL1	1p34.2	RERE*	1p36.23
RAG1AP1	1q22	PTPRF	1p34
ISG20L2	1q23.1	RALGPS2*	1q25.2
SH2D2A	1q21	H3F3A+	1q41
DCAF8	1q22–q23	PPP1CB+	2p23
GPR25	1q32.1	MAP4K3+	2p22.1
PROM2	2q11.1	CDK5R2*	2q35
CREB1	2q34	EOMES+	3p24.1
MFSD10	4p16.3	GRAMD1C	3q13.31
SH3RF2	5q32	GMPR	6p23
TNF*	6p21.3	ITPR3+	6p21
HSD17B8	6p21.3	SNX3*	6q21
C6orf108	6p21.1	FOXO3A+	6q21
TAGAP	6q25.3	CDK19*	6q21
NFE2L3	7p15.2	EIF3B*	7p22.3
GSTK1	7q35	IMPAD1	8q12.1
PDIA4	7q35	ADRB1+	10q24–q26
RAB11FIP1	8p11.22	TALDO1*	11p15.5–p15.4
RPL8	8q24.3	TTC9C	11q12.3
DUSP5	10q25	SAC3D1	11q13.1
RPLP2	11p15.5	FGF19+	11q13.1
C14orf70	14q32.2	PDS5B*	13q12.3
PSTPIP1	15q24–q25.1	RBM26	13q31.1
RHCG	15q25	MOAP1*	14q32
IL32	16p13.3	BTBD6	14q32
CIITA	16p13	RYR3+	15q14–q15
ALKBH5	17p11.2|17p11.2	TRPM7+	15q21
C17orf58	17q24.2	PDF	16q22.1
9-Sep	17q25	ITGB3+	17q21.32
LONP1	19p13.2	HLF*	17q22
TNFSF14	19p13.3	DGKE*	17q22
TNFSF14	19p13.3	TIMP2+	17q25
APG4D	19p13.2	PIP5K1C+	19p13.3
ZBTB32	19q13.1	RTN2-FLJ40125	19q13.32
FBXO17	19q13.2	U2AF2*	19q13.42
FAM83E	19q13.33	TRIM28+	19q13.4
SRXN1	20p13	FKBP1A+	20p13
CPXM1	20p13–p12.3	GALR3	22q13.1
YWHAH	22q12.3	CBX7+	22q13.1
CENPM	22q13.2		
TCF20	22q13.3|22q13.3		

* Genes detected in IPA to generate the 2 pathways in Fig. 5

**Fig. 4:**
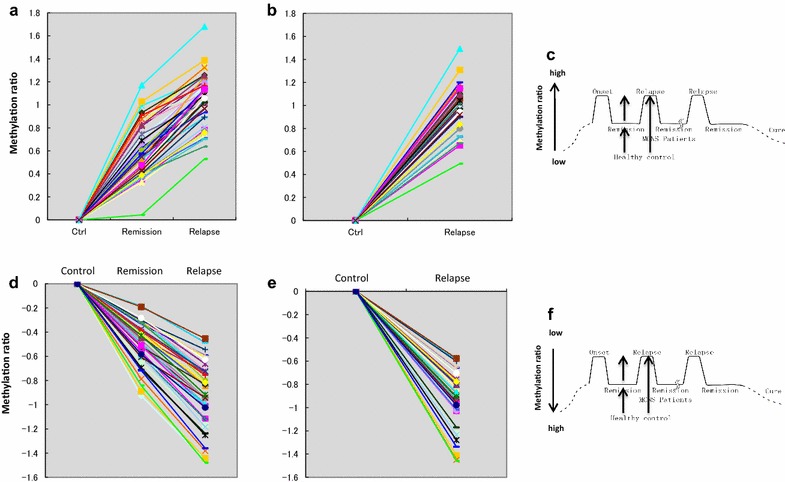
DNA methylation changes in 82 of 85 genes detected in Th0s were in accordance with disease activity. Among the 85 genes co-occurring in Th0s, 82 showed changes in methylation status according to disease activity. Forty of the 82 genes showed significantly higher methylation at relapse compared to remission (**a**) and controls (**b**), or at remission compared to controls (**a**) in each comparison. Additionally, the remaining 42 of the 82 genes were always less methylated at relapse compared to remission (**d**) and controls (**e**), or at remission compared to controls (**d**) in each comparison. The difference between control and relapse (**b** and **e**) was comparable to the sums of differences between control to remission and remission to relapse (**a** and **d**) at every probe of 82 genes, both in increasing and decreasing methylation towards relapse, as shown in **c** and **f**. The *line drawn in the same color* indicates the methylation ratio obtained from the same probe spotted in each array for 3 comparisons (**a**, **b**, **d**, and **e**)

Among the 82 genes, the degree of methylation in 40 genes was higher in relapse than in remission (Fig. 4a) and controls (Fig. 4b), and higher in remission than in controls (Fig. 4a), i.e. relapse > remission > controls. The difference between the control and relapse (Fig. 4b) was comparable to the sum of the differences between the control to remission and remission to relapse (Fig. 4a, see c for the concept). The remaining 42 genes were less methylated in relapse than in remission (Fig. 4d) and controls (Fig. 4e) and less methylated in remission than in controls (Fig. 4d), i.e. relapse < remission < controls. Signal intensity ratios between relapse and controls (Fig. 4e) showed similar cumulative differences between relapse to remission and remission to controls (Fig. 4d, see f for the concept), indicating the accuracy of the 3 different experiments comparing relapse versus remission, relapse versus control, and remission versus control (Additional file 1: Table S1). The remaining 3 genes from the 85 genes detected in Th0s (Additional file 1: Table S1) and the 4 co-occurring genes in monocytes showed no specific changes in DNA methylation in association with disease activity (data not shown).

### Ingenuity pathway analysis (IPA) of 85 co-occurring genes in Th0s

**Fig. 5:**
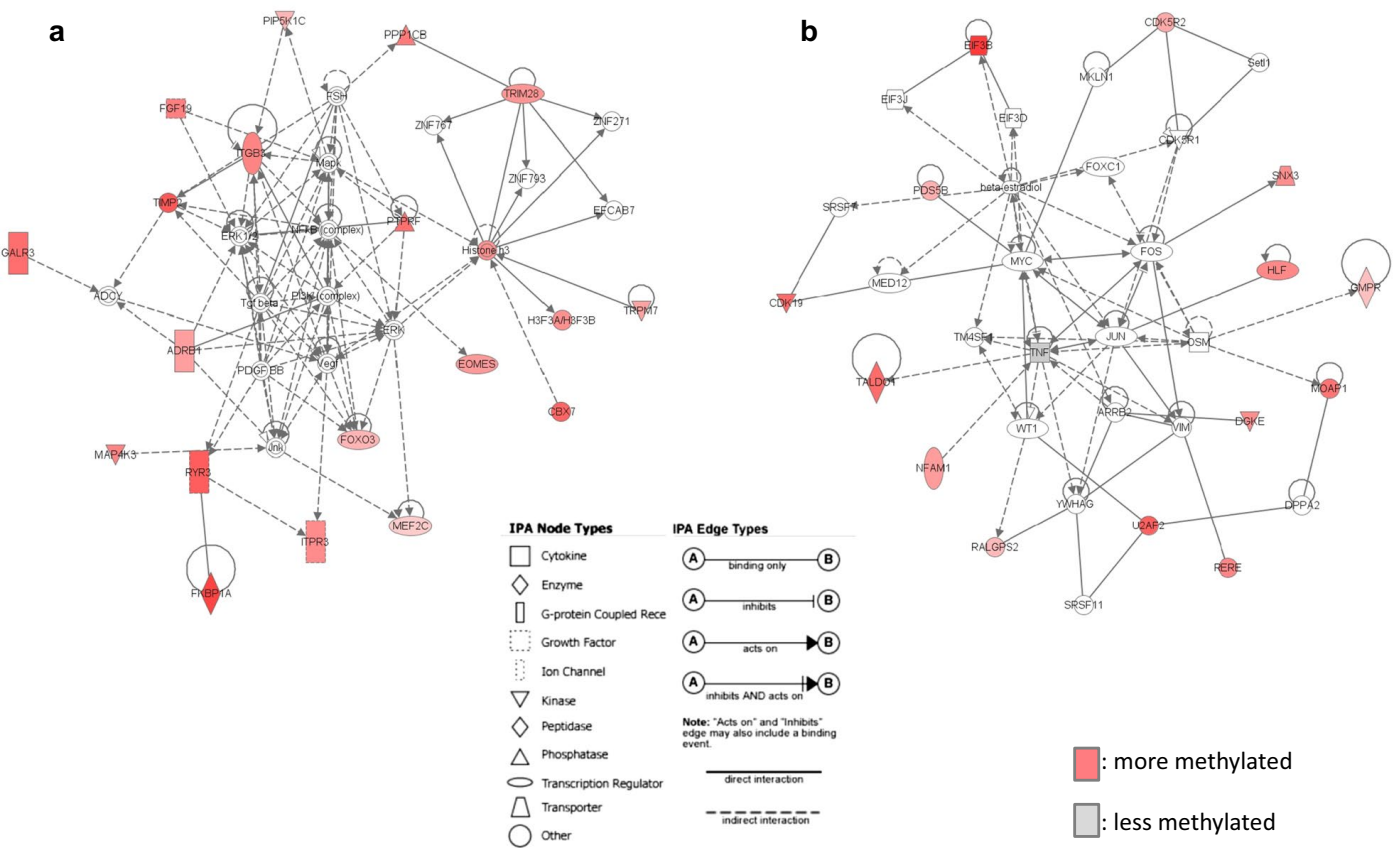
Two networks diagrams generated by ingenuity pathway analysis with the 85 genes. The associated network functions of network 1 (**a**) were cell-mediated immune response, cellular development, and cellular function and maintenance and included 19 of the 85 genes, with a score of 47, and those of network 2 (**b**) were gene expression, cellular development, and cellular growth and proliferation, and included 15 of the 85 genes, with a score of 31. The networks are based on known protein–protein interactions and functional relationships. The intensity of gene (node) *color in the networks* indicates the degree of hypermethylation (*red*) or hypomethylation (*gray*). Nodes are displayed using *various shapes* that represent the functional class of gene products

The 85 co-occurring genes in Th0s were analyzed by IPA [16, 17], which generated 2-network diagrams. The associated functions of the genes in network 1, which contained 19 of the 85 genes, included cell-mediated immune response, cellular development, and cellular function and maintenance (Fig. 5a), with a score of 47. Genes in network 2, which contained 15 of the 85 genes, had roles in gene expression, cellular development, and cellular growth and proliferation (Fig. 5b), with a score of 31. Top molecules and top canonical pathways detected by IPA are shown in Table 4.

**Table 4:** Ingenuity pathway analysis of the 85 co-occurring genes

(a) Top molecules			
Genes	Description	Chromosome	Exp. value
SAC3D1	SAC3 domain containing 1	11q13.1	0.772
EIF3B	Eukaryotic translation initiation factor 3, subunit B	7p22.3	0.692
PDF	Peptide deformylase (mitochondrial)	16q22.1	0.680
FKBP1A	FK506 binding protein 1A, 12 kDa	20p13	0.661
TIMP2	TIMP metallopeptidase inhibitor 2	17q25	0.586
RYR3	Ryanodine receptor 3	15q14-q15	0.567
U2AF2	U2 small nuclear RNA auxillary factor 2	19q13.42	0.558
B3GALT6	UDP-Gal:betaGal beta 1, 3-galactosyltransferase polypeptide 6	1p36.33	0.543
CBX7	Chromobox homolog 7	22q13.1	0.536
CDX19	Cyclin-dependent kinase 19	6q21	0.523

(b) Top canonical pathways		
Name	P value	Ratio
Insulin receptor signaling	3.45E−03	3/139 (0.022)
Protein kinase A signaling	5.74E−03	4/327 (0.012)
Clarin-mediated endocytosis signalling	6.29E−03	3/172 (0/017)
Spliceosomal cycle	7.06E−03	1/10 (0.100)
Calcium signaling	7.93E−03	3/207 (0.014)

### Co-occurring genes between Th0s isolated from patients in relapse and remission, and healthy controls

We analyzed 206 genes that were common in the 2 comparisons, including in relapse versus remission and relapse versus controls (Additional file 2: Table S2), and 58 genes in relapse remission versus controls (Additional file 3: Table S3) in Th0 cells, shown in the Venn diagram in Fig. 3b. The former 206 genes are proteinuria-related genes, while the latter 58 genes are disease-related genes. Among the 206 co-occurring genes, 186 genes were less methylated in the relapse (proteinuric) state than in the remission and control groups (non-proteinuric state), while the remaining 20 were more methylated in the relapse (proteinuric) state than in other samples (non-proteinuric) (Additional file 2: Table S2). Furthermore, among the latter 58 co-occurring genes, 20 were less methylated in both relapse and remission (disease) than in controls (non-disease), while the remaining 37 genes were more methylated in relapse and remission (disease) than in controls (non-disease) (Additional file 3: Table S3). The consistency of the results from the 2 different experiments using each probe indicates the accuracy of the experiment and the high probability of detection. We further examined these genes using DAVID bioinformatics resources 6.7 [18]. The 5 KEGG pathways identified the 206 proteinuria-associated genes, cytokine-cytokine receptor interaction (hsa04060), and hematopoietic cell lineage (hsa04640), glycine, serine, and threonine metabolism (hsa00260), and 56 disease-associated genes, including 1 KEGG pathway of focal adhesion (hsa04510) (Additional file 4: Table S4, Table S5).

## Discussion

**Fig. 6:**
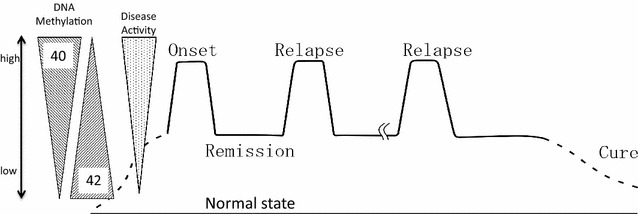
Scheme of correlation between DNA methylation change in 82 genes and disease activity. DNA methylation of 82 genes identified in Th0s varied according to disease activity, i.e., relapse, remission, or healthy, in 42 genes increasing and in 40 genes decreasing DNA methylation in relapse

By analyzing DNA methylation in Th0s and monocytes sorted from peripheral blood mononuclear cells (PBMCs) isolated from MCNS patients and healthy controls, we successfully identified 82 genes in Th0s whose DNA methylation levels varied according to disease activity, i.e., relapse, remission, or healthy (Fig. 6). However, in monocytes, only 4 genes were co-occurring. It has long been implicated that helper T cells rather than monocytes are involved in MCNS pathogenesis [1–4]. The current results suggest that the epigenetic status of helper T cells is altered and is associated with MCNS pathogenesis. DNA methylation analysis possibly enabled the identification of relevant genes associated with disease activity, particularly between remission and healthy controls, despite situations in which patients showed no symptoms and no proteinuria, such that their physical status was similar to that of healthy controls. Genes were less likely to be detected in the expression analysis when they caused similar phenotypes and clinical symptoms.

We previously identified 3 genes (*GATA2*, *PBX4*, and *NYX*) showing significantly lower methylation in Th0s in the relapse state than in the remission state by using regression analysis [14], which was originally used in the MIAMI method [19, 20]. In the current study, we used the Mahalanobis distance rather than regression analysis and determined that probes showing 99% CI were significant. Mahalanobis distance is the distance of the sample points from the center of the mass. Therefore, this value shows greater correlation with the differences in DNA methylation ratios between 2 samples in paired comparisons compared to regression analysis. The distributions of distance for all probes from the center of the mass were significantly different (*P* < 0.0001) between monocytes and Th0s, even though they were from the same individuals, and were significantly greater in Th0s than in monocytes in all 3 comparisons (Fig. 2a–c). This indicates that the steady-state regulation of DNA methylation differs between monocytes and Th0s and that more significant changes in DNA methylation were observed in Th0s than in monocytes in each comparison.

MIAMI is used for semi-quantitative and comparative analysis of DNA methylation. In 82 (95.6%) of the 85 co-occurring genes in 3 permutations of 3 Th0s samples, DNA methylation changes occurred in the same direction and was consistent in all 3 comparisons, and these changes were in accordance with the level of disease activity. This finding indicates that DNA methylation of the 82 genes is very relevant to MCNS development. Furthermore, 2 associated network functions were identified by IPA, involving 34 of the 85 co-occurring genes. The network functions contained nuclear factor kappa B (NF-κB) (Fig. 5a) and tumor necrosis factor alpha (TNF-α) (Fig. 5b) networks, both of which have been previously implicated in MCNS pathogenesis [21–25]. Particularly, *TNF* was less methylated in relapse than in remission, which is consistent with previous reports of elevated TNF-α levels in relapse [25, 26]. Moreover, the proteinuric state was associated with 206 co-occurring genes, while the disease was associated with 58 co-occurring genes, demonstrating that the direction of changes in DNA methylation in Th0s was consistent with disease activity (Fig. 3b) (Additional file 2: Table S2, Additional file 3: Table S3).

In this study, we focused on immunocytes and their epigenetic states. We purified and used 2 different cell types from PBMCs isolated from the participants: monocytes, which are precursors of antigen-presenting cells derived from the myeloid cell series, and Th0s, derived from the lymphoid system, because different types of blood cells have different methylation patterns that reflect differences in gene expression during cell differentiation into specific lineages [14]. An increasing body of evidence suggests that epigenetic regulation is essential for the differentiation of blood cells such as T helper type 2 (Th2) cells [27, 28]. Therefore, we isolated and studied monocytes and Th0s rather than total PBMCs, which contain a mixture of various cell types in different developmental stages and in differing epigenetic states. This restricted the analysis to epigenetic changes related to disease development, avoiding the inclusion of changes caused by differences in cell composition in the analysis with isolated cell types. Although Th2 activation has been shown to be involved in MCNS [21, 29–32] we hypothesized that epigenetic changes occur in Th0s before differentiation into Th2 cells, predisposing individuals to the active state of the disease. Examination of Th0s revealed that relevant genes showed modified DNA methylation states associated with disease activity. This is consistent with the results of a previous study showing that immature lymphocytes play a role in MCNS pathogenesis [33].

We found that variations in DNA methylation are associated with disease activity, proteinuria, and disease development in Th0s rather than in monocytes. Furthermore, the functions of the 82 genes associated with disease activity were found to be related to the TNF-α or NF-κB pathways, which have been reported to be related to MCNS pathogenesis. These results may suggest that the DNA methylation changes in Th0s were not only concomitant with disease activity, but also functionally relevant.

The limitation of this study is that limited number of patients and heterogeneity in treatment states were included. Genes that are sensitive to prednisolone and cyclosporine exposure in DNA methylation may be included in the detected genes. Further studies that include a larger number of MCNS patients without any immunotherapies both at relapse and remission are required for the identification of candidate genes for MCNS.

## Conclusions

Based on our data though it is preliminary, regulation of DNA methylation in Th0s may underlie the pathogenesis and pathophysiological states of MCNS. This suggests that DNA methylation analysis could be potentially a useful diagnostic tool that can be used to distinguish different disease states, particularly between remission and cured states.

## Methods

### Patients

Samples for MIAMI analysis were obtained from 6 male patients with MCNS (Table 1) at relapse and following complete remission. All patients had developed nephrotic syndrome before 16 years of age, and were diagnosed according to the criteria of the International Study of Kidney Disease in Children. In brief, the diagnostic criteria for MCNS are (1) heavy proteinuria of ≥0.96 g/day m^2^ BSA, determined using a urine sample collected overnight; (2) hypoalbuminemia of ≤2.5 g/dL; (3) no evidence of underlying systemic diseases nor exposure to agents that are known to be associated with NS at its onset; (4) response during 8 week of the initial treatment with 60 mg/24 h m^2^ prednisone in three divided daily doses for 4 week, followed by administration of 40 mg/24 h m^2^ in divided doses on 3 consecutive days per week for 4 week. Response was defined as the reduction in the rate of urinary excretion of protein to <4 mg/h m^2^ (Albustix, 0 to trace) for 3 consecutive days. Relapse involved the reappearance of proteinuria of 40 mg/h m^2^ (Albustix, + +or greater) for 3 consecutive days [15]. A frequent relapser is a patient who experiences 2 or more relapses during a 6-month period subsequent to treatment [15].

Informed consent was obtained from all parents for the children and from the patients who were older/adolescents. The healthy controls included five male voluntary participants who were 23 years old. This study was approved by the Ethics Committee of Gunma University Graduate School of Medicine, Japan (Receipt Number 89).

### Cell separation

PBMCs were isolated from 20 mL samples of anti-coagulated blood obtained by gradient separation using the Lymphprep™ Tube (Axis-Shield PoC AS, Oslo, Norway). Monocytes and Th0s were separated using an autoMACS Pro Separator (Miltenyi Biotec, Bergisch Gladbach, Germany) from PBMCs that had been magnetically labeled with CD14, CD4, and CD45RO MicroBeads (Miltenyi Biotec). To obtain monocytes, the CD14-positive (CD14^+^) fraction was collected as monocytes, whereas CD14-negative, CD4-positive, and CD45RO-negative (CD14^−^CD4^+^CD45RO^−^) fractions were collected as Th0s. Flow cytometric analysis using a MACSQuant^®^ Analyzer (Miltenyi Biotec) showed that the precision of cell separation was 96.2% for CD14^+^ cells and 94.46% for CD4-positive and CD45RA-positive cells as a CD45RO-negative fraction [14].

CD14^+^ monocytes were obtained from all 6 patients, while CD14^−^CD4^+^CD45RO^−^ Th0 cells were obtained from 4 of these patients (Table 1a) for MIAMI analysis, both at relapse and following complete remission. Genomic DNA (gDNA) was extracted from these cells as previously described [34]. Consistent amounts of extracted gDNA (300 ng from monocytes and 250 ng from Th0s) from each selected cell type were pooled and used for subsequent DNA methylation analysis using MIAMI. This was done to exclude the influence of epigenetic differences among cell types, identify specific changes resulting from the clinical courses of MCNS, and reduce noise caused by individual differences.

### MIAMI analysis

To determine methylation states, we used the MIAMI method, which provides high-throughput global analysis of DNA methylation [19, 20]. Since this method is based on the comparison of the digestion efficiency between 2 different samples by methyl-sensitive *Hpa*II restriction enzymes, it indicates the differences in DNA methylation between the samples. The method was performed as described previously using 1.8 and 1.0 µg of pooled gDNA from the monocytes of 6 patients and Th0s of 4 patients, respectively [14, 19, 20]. Briefly, isoschizomers (*Hpa*II and *Msp*I) that recognize the same recognition site (CCGG) were used. Pooled gDNA was digested with *Hpa*II, a methylation-sensitive restriction enzyme that cleaves only unmethylated DNA, and then the samples were adapter-ligated and amplified by PCR using primers designed against the adapter sequences. The samples were further digested with *Msp*I, a methylation-insensitive enzyme that digests CCGG sites irrespective of their methylation status, and were amplified again with the same primer set (*Hpa*II–*Msp*I treatment). The second *Msp*I treatment yielded amplicons from unmethylated DNA fragments only. Hence, only *Hpa*II-cleavable unmethylated DNA fragments were amplified and were quantified based on their fluorescence intensities by microarray analysis. The amplified products were then labeled with Cy3 (remission or control samples) or Cy5 (relapse or remission samples) and co-hybridized (i.e. remission vs relapse, control vs relapse, or control vs remission) to a microarray spotted with 38,172 sixty-mer oligonucleotides covering the vicinity of the transcription start sites of 14,978 genes. Following hybridization, the microarray was scanned and the fluorescence intensities obtained were quantified and normalized. The same pooled gDNA samples were treated first with *Msp*I rather than *Hpa*II (*Msp*I–*Msp*I treatment) and analyzed on a duplicate array to correct for false-positives caused by single-nucleotide polymorphisms or incomplete digestion.

### IPA

Gene networks and canonical pathways representing key genes were identified using the curated IPA database (http://www.ingenuity.com/). The data set containing gene identifiers and corresponding fold-changes was uploaded into the web-delivered application, and each gene identifier was mapped to its corresponding gene object in the Ingenuity Pathways Knowledge Base. IPA generates hypothetical protein–protein interactions, which are not limited to “binding”, but also include “activation”, “inhibition”, “expression”, and other interactions described in the literature. Functional analysis identified the biological functions and/or diseases that were most significant to the data sets. Fisher’s exact test was performed to calculate a *P* value to indicate the probability that each biological function and/or disease assigned to the data set was because of chance alone. The data set was mined for significant pathways using the IPA library of canonical pathways, using either (1) a ratio of the number of genes from the data set that mapped to the pathway divided by the total number of genes that mapped to the canonical pathway or (2) a Fisher’s exact test to calculate a *P* value determining the probability that the association between the genes in the data set and the canonical pathway was explained by chance alone [19, 20].

### DAVID bioinformatics resources

DAVID bioinformatics resources (http://david.abcc.ncifcrf.gov) consist of an integrated biological knowledgebase and analytic tools aimed at systematically extracting biological meaning from large gene/protein lists. DAVID is a high-throughput and integrated data-mining environment that can analyze gene lists derived from high-throughput genomic experiments. The procedure first requires uploading a gene list containing any number of common gene identifiers, followed by analysis using one or more text and pathway-mining tools such as gene functional classification, functional annotation chart or clustering, and a functional annotation table. The major statistical methods have been described previously [18], and Additional file 2: Table S2 shows the associated parameters used in DAVID.

### Statistical analysis

Mahalanobis distance was calculated using R version 2.0. The threshold values were determined based on 99% confidence intervals (Fig. 1). Statistical analyses of the distance distributions from the center of mass (Mahalanobis distance) for each probe used in the assays were performed using a non-parametric Mann–Whitney *U* test (Fig. 2). Statistical analyses were performed using GraphPad PRISM5 software (GraphPad Software, La Jolla, CA, USA) with the significance level set at *P* < 0.05.

## Additional files

**Additional file 1.** MIAMI results of 85 co-occurring genes in Th0s identified in all 3 comparisons.

**Additional file 2.** MIAMI results of 206 co-occurring genes in Th0s identified in the 2 comparisons; relapse versus remission and relapse versus controls (proteinuria-related genes).

**Additional file 3.** MIAMI results of 58 co-occurring genes in Th0s identified in the 2 comparisons; relapse versus controls and remission versus controls (disease-related genes).

**Additional file 4.** **Table S4:** Analysis with DAVID bioinformatics resources: Analysis of the 206 proteinuria oriented co-occurring genes. **Table S5:** Analysis with DAVID bioinformatics resources: Analysis of the 58 disease-related co-occurring genes.
