# Supplementary material for: Changes in DNA methylation in naïve T helper cells regulate the pathophysiological state in minimal-change nephrotic syndrome
Source: BMC Res Notes. 2017 Sep 15;10:480. doi: 10.1186/s13104-017-2719-1 (PMC5603023; doi:10.1186/s13104-017-2719-1)
Supplement: Supplementary file 4 — Additional file 4. Table S4: Analysis with DAVID bioinformatics resources: Analysis of the 206 proteinuria oriented co-occurring genes. Table S5: Analysis with DAVID bioinformatics resources: Analysis of the 58 disease-related co-occurring genes. [file 13104_2017_2719_MOESM4_ESM.docx]

Table S4 Analysis with DAVID bioinformatics resources: Analysis of the 206 proteinuria oriented co-occurring genes

| Category | Term | Count | % | P Value | Genes | List Total | Pop Hits | Pop Total | Fold Enrichment | Bonferroni | Benjamini | FDR |
| --- | --- | --- | --- | --- | --- | --- | --- | --- | --- | --- | --- | --- |
| KEGG_  PATHWAY | hsa03010:  Ribosome | 9 | 4.663212435 | 3.88E-05 | RPL18,  RPL19,  RPL18A,  RPL9,  RPL3,  RPS10,  RPL38,  RPS2,  RPS8 | 77 | 87 | 5085 | 6.831616659 | 0.003560681 | 0.003560681 | 0.042348908 |
| KEGG_  PATHWAY | hsa04060:  Cytokine-cytokine receptor interaction | 10 | 5.18134715 | 0.015368276 | LIF,  TNFRSF9,  IL3,  IL2RB,  CCR6,  TNF,  CXCR5,  TNFRSF4,  IL17RB,  FLT3LG | 77 | 262 | 5085 | 2.520571032 | 0.759457498 | 0.509548676 | 15.56593877 |
| KEGG_  PATHWAY | hsa04640:  Hematopoietic cell lineage | 5 | 2.590673575 | 0.03896913 | IL3,  TNF,  ITGA3,  CD5,  FLT3LG | 77 | 86 | 5085 | 3.839474479 | 0.974187201 | 0.704463107 | 35.22485845 |
| KEGG_  PATHWAY | hsa00260:  Glycine, serine, and threonine metabolism | 3 | 1.554404145 | 0.077608508 | CHDH,  SHMT2,  AOC3 | 77 | 31 | 5085 | 6.390867197 | 0.999408157 | 0.844026206 | 58.62809428 |
| KEGG_  PATHWAY | hsa05010:  Alzheimer's disease | 6 | 3.10880829 | 0.095635794 | TNF,  NDUFA6,  COX4I2,  ATP5F1,  ITPR3,  CAPN2 | 77 | 163 | 5085 | 2.430882001 | 0.999903708 | 0.842703913 | 66.65284954 |

Table S5 Analysis with DAVID bioinformatics resources: Analysis of the 58 disease-related co-occurring genes

| Category | Term | Count | % | P Value | Genes | List Total | Pop Hits | Pop Total | Fold Enrichment | Bonferroni | Benjamini | FDR |
| --- | --- | --- | --- | --- | --- | --- | --- | --- | --- | --- | --- | --- |
| KEGG_  PATHWAY | hsa04510:  Focal adhesion | 5 | 0.326370757 | 0.07695447 | CCND3,  PIP5K1C,  ZYX,  ITGB3,  PPP1CB | 42 | 201 | 5085 | 3.011727079 | 0.998475691 | 0.998475691 | 57.40373944 |
